# Group analysis and classification of working memory task conditions using electroencephalogram cortical currents during an n-back task

**DOI:** 10.3389/fnins.2023.1222749

**Published:** 2023-10-24

**Authors:** Shinnosuke Yoshiiwa, Hironobu Takano, Keisuke Ido, Mitsuo Kawato, Ken-ichi Morishige

**Affiliations:** ^1^Graduate School of Engineering, Toyama Prefectural University, Imizu, Japan; ^2^Department of Intelligent Robotics, Toyama Prefectural University, Imizu, Japan; ^3^Center of Liberal Arts and Science, Toyama Prefectural University, Imizu, Japan; ^4^Brain Information Communication Research Laboratory Group, Advanced Telecommunications Research Institute International, Kyoto, Japan; ^5^Neural Information Analysis Laboratories, Advanced Telecommunications Research Institute International, Kyoto, Japan

**Keywords:** working memory, EEG, hierarchical Bayesian estimation, sparse logistic regression, artifact

## Abstract

Electroencephalographic studies of working memory have demonstrated cortical activity and oscillatory representations without clarifying how the stored information is retained in the brain. To address this gap, we measured scalp electroencephalography data, while participants performed a modified n-back working memory task. We calculated the current intensities from the estimated cortical currents by introducing a statistical map generated using Neurosynth as prior information. Group analysis of the cortical current level revealed that the current amplitudes and power spectra were significantly different between the modified n-back and delayed match-to-sample conditions. Additionally, we classified information on the working memory task conditions using the amplitudes and power spectra of the currents during the encoding and retention periods. Our results indicate that the representation of executive control over memory retention may be mediated through both persistent neural activity and oscillatory representations in the beta and gamma bands over multiple cortical regions that contribute to visual working memory functions.

## 1. Introduction

Although the human brain can temporarily store information, such as numbers or strings, it remains unclear how the stored information is retained in the brain (Postle, 2006; D’Esposito and Postle, 2015; Constantinidis and Klingberg, 2016; Chai et al., 2018).

Baddeley’s model of working memory consists of one central executive and three subsystems: the phonological loop, the visuospatial sketchpad, and the episodic buffer. The phonological loop stores verbal information and revives auditory memory. A visuospatial sketch pad is a storage system that holds and processes non-verbal information. An episodic buffer is a temporary storage system that integrates visual, spatial, and verbal information with time sequencing. The central executive acts as a supervisory system and controls the flow of information from and to its subsystems, thus focusing on and dividing attention and switching and activating long-term memory to support goal-oriented behavior (Baddeley and Hitch, 1974; Baddeley, 2010).

Human functional magnetic resonance imaging (fMRI) studies have shown that the prefrontal and anterior cingulate cortices play major roles in implementing the concept of working memory (Osaka et al., 2003; Postle, 2015). Electroencephalography (EEG) and magnetoencephalography (MEG) studies have demonstrated that oscillatory activity is related to working memory content and load (Sarnthein et al., 1998; Miltner et al., 1999). Miller et al. proposed a model in which executive control acts via the interplay between gamma network oscillations in superficial cortical layers and alpha and beta oscillations in deep cortical layers (Lundqvist et al., 2016; Miller et al., 2018). However, how Baddeley’s psychological model (particularly the representation of the executive control involved in memory retention) is implemented in the nervous system remains an open question.

fMRI has been widely used in working memory studies in humans. This method, which has the advantage of high spatial resolution, can be used to identify brain regions related to working memory and investigate their functional connectivity. However, fMRI cannot acquire high-resolution temporal data due to its measurement principles.

However, EEG and MEG are candidates for recording high-resolution temporal data used for brain activity. EEG/MEG studies on working memory have demonstrated cortical activity and oscillatory representations. However, it is difficult to use the EEG method to acquire high-resolution spatial data because of volume conduction effects and large interelectrode distances. MEG has a significant advantage over EEG because magnetic fields pass through the head without distortion; however, a higher spatial resolution is required. Moreover, a visual stimulus may cause task-related eye movements that induce eye artifacts in the EEG/MEG data. These eye artifacts have some correlation with brain activity, and separating the components of brain activity and artifact components is difficult using conventional statistical methods such as principal component analysis (PCA) or independent component analysis (ICA).

We simultaneously estimated both cortical currents and multiple extra-brain source currents from contaminated EEG/MEG data. Although the measured EEG/MEG data were contaminated by eye artifacts, the proposed method separated the effects of artifacts and estimated the cortical currents of the entire brain using the extra-dipole method (Morishige et al., 2014, 2021). The sparse logistic regression (SLR) method can automatically select, in a data-driven manner, truly important features of working memory calculated from the estimated cortical currents in multiple cortical regions (Yamashita et al., 2008). Furthermore, it can predict the task conditions of the working memory from selected current sources. In this study, by combining the extra-dipole method and SLR, we predicted working memory task conditions from brain regions and investigated the types of information represented in these cortical regions.

Two hypotheses have been proposed to explain the brain mechanisms used for memory retention in working memory, based on the following question: Is it a simple persistent spiking pattern or a periodic pattern of theta, alpha, beta, and gamma bandwidths? If memory retention is achieved by sustained firing patterns of neurons, some differences should exist in the intensity of the estimated current at each dipole. If the function is implemented in periodic patterns, the spectral features of the estimated currents will differ. We examined differences in the magnitudes of the estimated currents in response to different memory loads and found significant differences in the encoding and retention periods. Furthermore, the spectral features of beta and gamma waves were significantly different in several cortical regions.

## 2. Materials and methods

### 2.1. Participants

Fourteen adults [11 men and 3 women; aged 21–51 years, mean age = 31.6 ± 12.2 (standard deviation) years] took part in this study. All participants had normal or corrected-to-normal visual acuity. All participants participated in the EEG experiments. Five other participants also participated in the fMRI experiment; however, these data were not included in the study. All experiments were approved by the Ethics Committee of Toyama Prefectural University, the Safety Committee of the Advanced Telecommunications Research Institute International (ATR), and the Ethics Committee of the Hokuriku Health Service Association. All experiments were performed in accordance with approved guidelines and regulations. Written informed consent was obtained from each participant before the experiment.

### 2.2. EEG data collections

We continuously recorded EEG data using a 64-channel ActiveTwo EEG system (BioSemi, Amsterdam, Netherlands) with electrodes attached to a nylon cap based on the extended 10–20 international system. The participants sat on a comfortable chair 50 cm away from a 24-inch LCD monitor (60-Hz refresh rate) in an electromagnetically shielded room. We recorded an electrooculogram (EOG) from four electrodes located at the left and right temples and above and below the left eye. We recorded a neck electromyogram (EMG) using two electrodes placed on the left sternocleidomastoid muscle. We also recorded finger electromyograms (EMGs) by using two electrodes placed in tandem on the extensor digitorum muscles of the right arm. To verify the timing of the visual stimulus, we measured its onset on the screen using a photodiode. We used either the 2-Button Response Pad (Current Designs, Inc., Philadelphia, PA) or the BSGP815GY GamePad (Buffalo, Inc., Aichi, Japan) as a response box to obtain participants’ feedback and measure the response time. However, due to a malfunction of the response box, the response time could not be measured for the two participants.

### 2.3. Magnetic resonance imagining data collection

T1-weighted structural images were obtained using either a 3 T Siemens Magnetom Prisma Fit scanner (Siemens AG, Erlangen, Germany) or a Vantage Orian 1.5 T Magnetic resonance imagining (MRI) system (Canon Medical Systems, Ohtawara, Japan), with a magnetization-prepared rapid gradient-echo (MPRAGE) sequence. The scanning parameters of the Siemens Magnetom Prisma Fit were as follows: repetition time (TR), 2,300 ms; echo time (TE), 2.98 ms; flip angle, 9°; voxel size, 1 mm; number of slices, 208; matrix size, 256 × 256; and field of view, 256 × 256 mm. Those of the Vantage Orian were as follows: TR, 20 ms; TE, 4.00 ms; flip angle, 15°; voxel size, 0.5 mm; number of slices, 400; matrix size, 512 × 512; and field of view, 256 × 256 mm.

### 2.4. Task design and procedure

**Figure 1:**
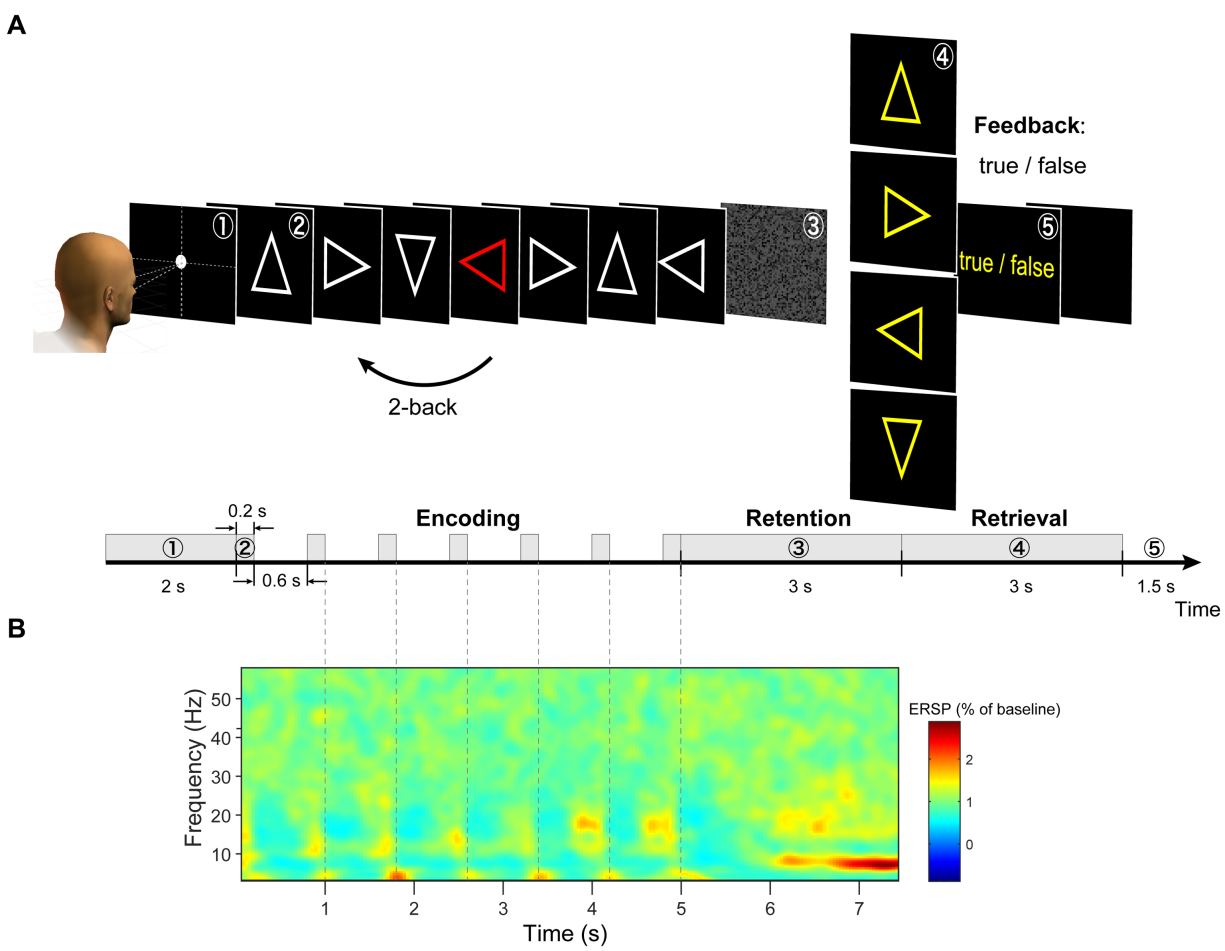
**(A)** Illustration of task design. **(B)** We extracted each trial from −0.5 to 8.0 s and calculated a grand average of the ERSP (event-related spectral perturbation) spectrogram of EEG signals across all channels (using wavelet analysis). During the retention period, the largest periodic change was observed between 6 and 7.5 s.

In the original version of the n-back task, figures were presented sequentially on the screen, and participants had to remember these sequences (Kirchner, 1958; WU-Minn HCP Consortium, 2015). This protocol is widely used; however, it poses difficulties for EEG data analysis in isolating brain activity during the encoding and retention periods. In this study, we modified the n-back working memory task. This task comprised three periods (Figure 1A). (a) During the encoding period, the modified 2-back task and delayed match-to-sample (DMTS) task were randomly presented. In the modified 2-back task, seven stimuli chosen from four types of arrows (left, right, up, and down) were presented and replaced sequentially on a monitor. One stimulus was randomly presented as a red arrow. Participants were instructed to memorize the direction of the arrow that appeared two steps before the red arrow. In the DMTS task, the serial presentation of a stimulus was the same as that in the modified 2-back task, except that a single-arrow stimulus chosen from the four types of arrows was used. The same arrow stimulus was presented seven times on a monitor. (b) Information is maintained for 3 s. A random pattern was presented to avoid visual aftereffects [Figure 1A (3)]. (c) During the retrieval period, the participants judged whether the probe arrow direction matched the retained direction by pressing one of the two buttons with their right index or middle finger [Figure 1A (4)]. The participants received visual feedback regarding the correctness of their responses [Figure 1A (5)].

The process comprised a single trial. Each session consisted of 20 trial repetitions, and each task consisted of eight sessions. Each participant performed 160 trials (20 trials × eight sessions). The order of the modified 2-back and DMTS tasks was counterbalanced across participants (left/right/up/down:36 trials; DMTS:16 trials). EEG and fMRI experiments were conducted on different days. The participants followed identical experimental protocols for the EEG and fMRI experiments.

### 2.5. EEG data analysis

We preprocessed the raw EEG data in the following steps using EEGLAB version 14.1.2 (Delorme and Makeig, 2004) running in MATLAB 2014b. The data were band-pass filtered in the range of 0.4–512 Hz (FIR filter of order 16,897; 0.2 Hz and 512.2 Hz cutoff frequencies (−6 dB); zero-phase) to remove the low-frequency drift components and the high-frequency noise components. Then, we applied a notch filter of 59–61 Hz to remove powerline noise (FIR filter of order 6761; 59.5 and 60.5 Hz cutoff frequencies; zero-phase). Next, the EEG data were downsampled from 2,048 to 512 Hz. We extracted single-trial EEG data epochs from −0.5 to 8.0 s with respect to the encoding onset (Figure 1B). After the extraction, we corrected the baselines to the pre-stimulus period (−0.5 to 0 s). During all sessions, noisy channels due to poor electrode contact and broken electrodes were identified by visual inspection and excluded. The data were re-referenced using the average reference (the reference signal was the average of all the electrodes). Signal deviations in the vertical EOG channel of more than 350 μV within the retention period were identified as eyeblinks. Signal deviations in all EEG channels of more than 200 μV within a retention period were identified as large artifacts. Trial data contaminated with eyeblinks and large artifacts were excluded from the analysis. Trials with incorrect responses during the retrieval period were excluded from the analysis. The remaining trials accounted for 79.6% of the total trials (Supplementary Table S1) and were used for the data analysis.

### 2.6. Meta-analysis fMRI prior

**Figure 2:**
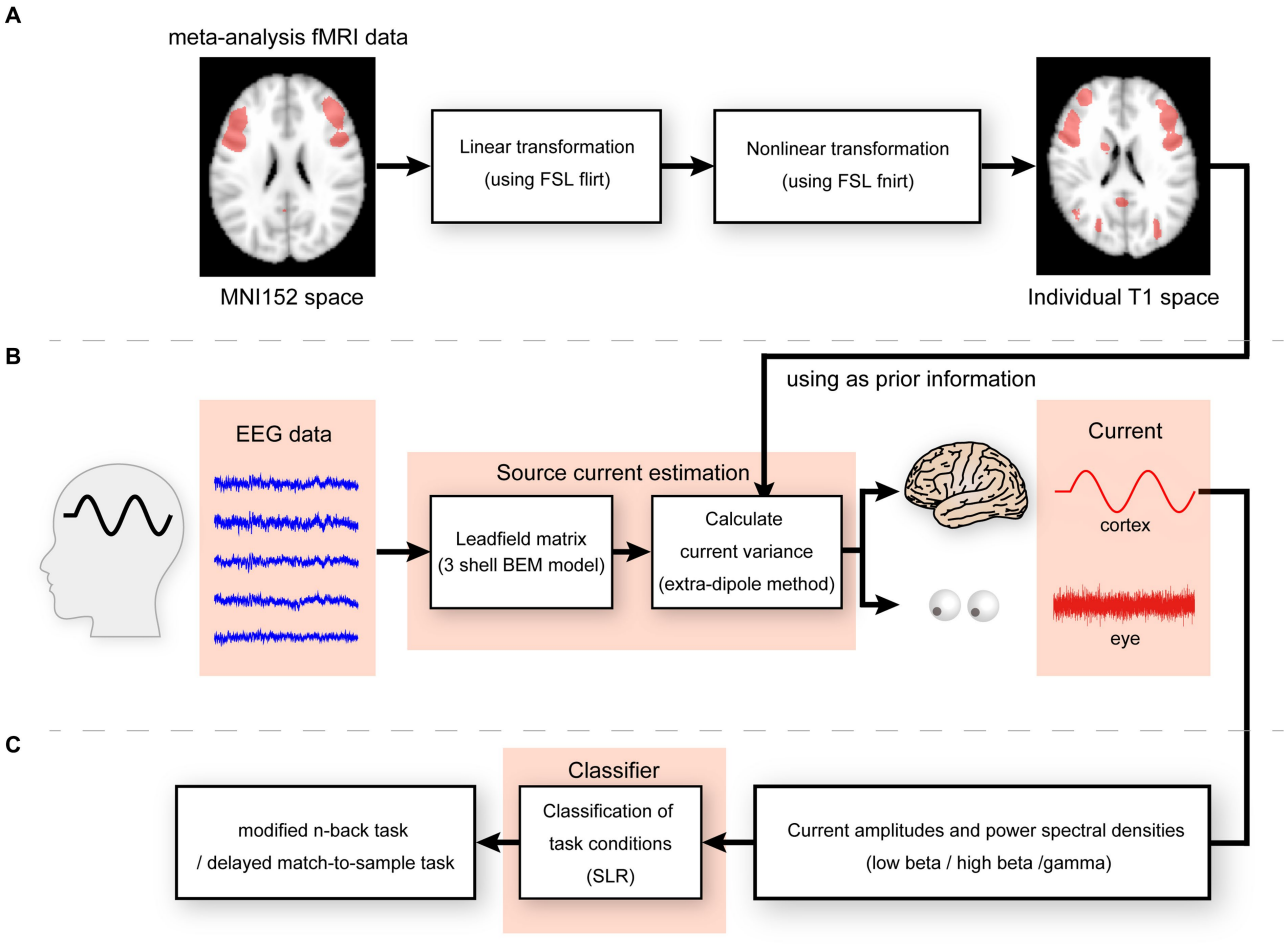
**(A)** Linear and non-linear transformation for meta-analysis fMRI data from MNI152 to individual T1 spaces. **(B)** Illustration of the extra-dipole method. **(C)** Illustration of classification of modified n-back and DMTS task conditions.

We generated a meta-analysis statistical map synthesized by Neurosynth (Yarkoni et al., 2011)^1^ by selecting the term “working memory” to express functional activities during the n-back task. After generation, the statistical map was co-registered to the participant’s structural image using the FSL tools FLIRT and FNIRT (Smith et al., 2004; Figure 2A). As the synthesized meta-analysis maps were defined on voxels, they were transformed into cortical surfaces using an inverse-distance weighted interpolation method. An imported map was used to calculate the parameters in the probability distribution of the prior current variances for hierarchical Bayesian estimation according to a previously established method (Suzuki and Yamashita, 2021).

### 2.7. Head and source models

We constructed a polygon cortical surface model for all participants using the FreeSurfer software (version 6.0.0; http://surfer.nmr.mgh.harvard.edu/; Dale et al., 1999) with a T1-structural image for each participant. The number of cortical surface dipoles in the participants was 10,004. The cortical current sources were located at the vertex points of the cortical surface model, and current sources were oriented perpendicular to the cortical surface. A positive current was defined as the one directed toward the interior of the cortex. The main noise source for the left and right eye movements was assumed to be the center of each eyeball. The position of each eyeball was obtained from the T1-structural images by visual inspection. Each extra-brain source was modeled using the resultant three-dimensional dipole current in the x–y–z direction. Six dipoles (two extra-brain sources × three directions) were located as described in our previous study (Morishige et al., 2014).

We used the three-shell boundary element method (BEM) derived from the MRI dataset (Mosher et al., 1999). The conductivities of the brain, skull, and skin were assumed to be 0.62, 0.03, and 0.62 S/m, respectively.

### 2.8. Cortical and extra-brain source current estimation

We calculated the cortical and extra-brain source currents using an extra-dipole method (Morishige et al., 2014) based on a hierarchical Bayesian method (Sato et al., 2004; Yoshioka et al., 2008) and simultaneously estimated the cortical and extra-brain source currents by placing the dipoles on both the cortical and extra-brain sources. This method can be used to estimate the cortical currents from EEG data contaminated with extra-brain sources (Figure 2B).

### 2.9. Group analysis for estimated cortical currents and oscillatory activities

Takeda et al. proposed a group analysis method for the time series of the estimated source currents (Takeda et al., 2019). We applied this method to examine the differences in the amplitudes and power spectra of the source currents estimated from EEG data.

We calculated the time series of trial-averaged source currents and scaled their amplitude, so they had a mean of 0 and a standard deviation of 1 in a baseline period (−0.5 to 0 s). The time series of the trial-averaged source currents was calculated from the normalized source currents for a single retention period. Then, we split the encoding and retention periods into 12 subperiods (0.2–1.0 s, 1.0–1.8 s, 1.8–2.6 s, 2.6–3.4 s, 3.4–4.2 s, 4.2–5.0 s, 5.0–5.5 s, 5.5–6.0 s, 6.0–6.5 s, 6.5–7.0 s, 7.0–7.5 s, and 7.5–8.0 s), and then, we compared all participants’ current amplitude in an encoding/retention subperiod between modified n-back and DMTS conditions with a paired *t*-test at each current source. To examine the differences in the spectral features of the two conditions, we estimated the power spectral density using Welch’s method for each source current in each trial in a baseline period and an encoding/retention subperiod from the estimated source currents and calculated the sum of power spectral densities in each frequency band of interest: theta (4–8 Hz), alpha (8–13 Hz), low beta (13–20 Hz), high beta (20–30 Hz), and gamma waves (30–50 Hz). We normalized the mean power spectral density of each frequency band using the baseline period values and converted them to a decibel scale using a log base (Cohen, 2014). We compared the normalized mean power spectral densities between the modified n-back and DMTS conditions using a paired t-test at each sampling time. The *p*-values for the paired t-test were corrected for multiple comparisons using Benjamini and Hochberg’s false discovery rate (FDR) procedure (Benjamini and Hochberg, 1995). The FDRs were controlled at 0.05.

### 2.10. Classification

To investigate the representation of working memory in cortical regions, we classified information on the task conditions of working memory using current amplitudes and power spectral densities during the encoding and retention periods. We selected 100 cortical dipole currents in the order of t-values generated by the Neurosynth meta-analysis statistical map and used them for classification. We computed the sum of the absolute current amplitudes and power spectral densities of the low beta, high beta, and gamma waves using Welch’s method. Sparse logistic regression was used to reduce the input dimensions of the current amplitudes and power spectral densities, which were then divided into two classes (modified n-back or DMTS tasks; Yamashita et al., 2008; Figure 2C) and evaluated using leave-one-out cross-validation. A permutation test was performed by randomizing the labels 100 times to determine whether the performance of the classifiers was statistically meaningful. The one-sided *p*-values of the test were calculated as the proportion of sampled permutations where the differences in means were greater than the test statistic. The accuracy, precision, recall, F-measure, and balanced accuracy were calculated and used for the evaluations. The p-values for the permutation test were corrected for multiple comparisons using Benjamini and Hochberg’s false discovery rate (FDR) procedure (Benjamini and Hochberg, 1995). The FDRs were controlled at 0.05.

The ratio of the trial numbers for the modified n-back and DMTS tasks was 144:16, which is a medium-imbalanced dataset. To address the imbalanced data problem in classification, we extended the original SLR and applied the formulation using weighted logistic regression (King and Zeng, 2001; Maalouf and Siddiqi, 2014). The likelihood function of the logistic regression can be rewritten as follows:


$$P\left(y|X,\beta \right)=\overset{N_{\mathrm{input}}}{\underset{i=1}{\prod }}\sigma_{i}^{w_{1}y_{i}}{\left(1-\sigma_{i}\right)}^{w_{0}\left(1-y_{i}\right)},$$

where $X=\left(x_{1},\cdots ,\;\;x_{N_{input}}\right)$ is an input feature vector , β is a weight vector including a bias term, y is the outcome vector (either $y_{i}=1$ or $y_{i}=0$ ), $w_{1}=N_{\mathrm{trial}\_\mathrm{all}}/\left(N_{\mathrm{class}}*N_{\mathrm{trial}\_\mathrm{DMTS}}\right)$ , $w_{0}=\left(N_{\mathrm{trial}\_\mathrm{all}}\right)/\left(N_{\mathrm{class}}*N_{\mathrm{trial}\_\mathrm{nback}}\right)$ , and $\sigma_{i}=1/\left(1+\exp\left(-x\right)\right)$ .

To improve computational efficiency, we used Z currents as cortical currents to calculate the sum of the current amplitudes and power spectral densities (Morishige et al., 2021).

## 3. Results

### 3.1. Behavior

All participants performed both modified 2-back and DMTS tasks with high success rates (mean success rate ± standard deviation, 94.3 ± 3.3% and 98.7 ± 2.7%, respectively). The response times for the two conditions were 0.83 ± 0.22 and 0.79 ± 0.24 s, respectively. There was no significant difference in response time [paired *t*-test: *t*(11) = 1.6657, *p* = 0.1240]. However, the success rate of the modified 2-back task was significantly lower than that of the DMTS condition [paired *t*-test: *t*(13) = 3.2412, *p* = 0.006], indicating that the EEG comparison among the different conditions could be influenced by the difficulty of the task.

### 3.2. Cortical and extra-brain source currents

**Figure 3:**
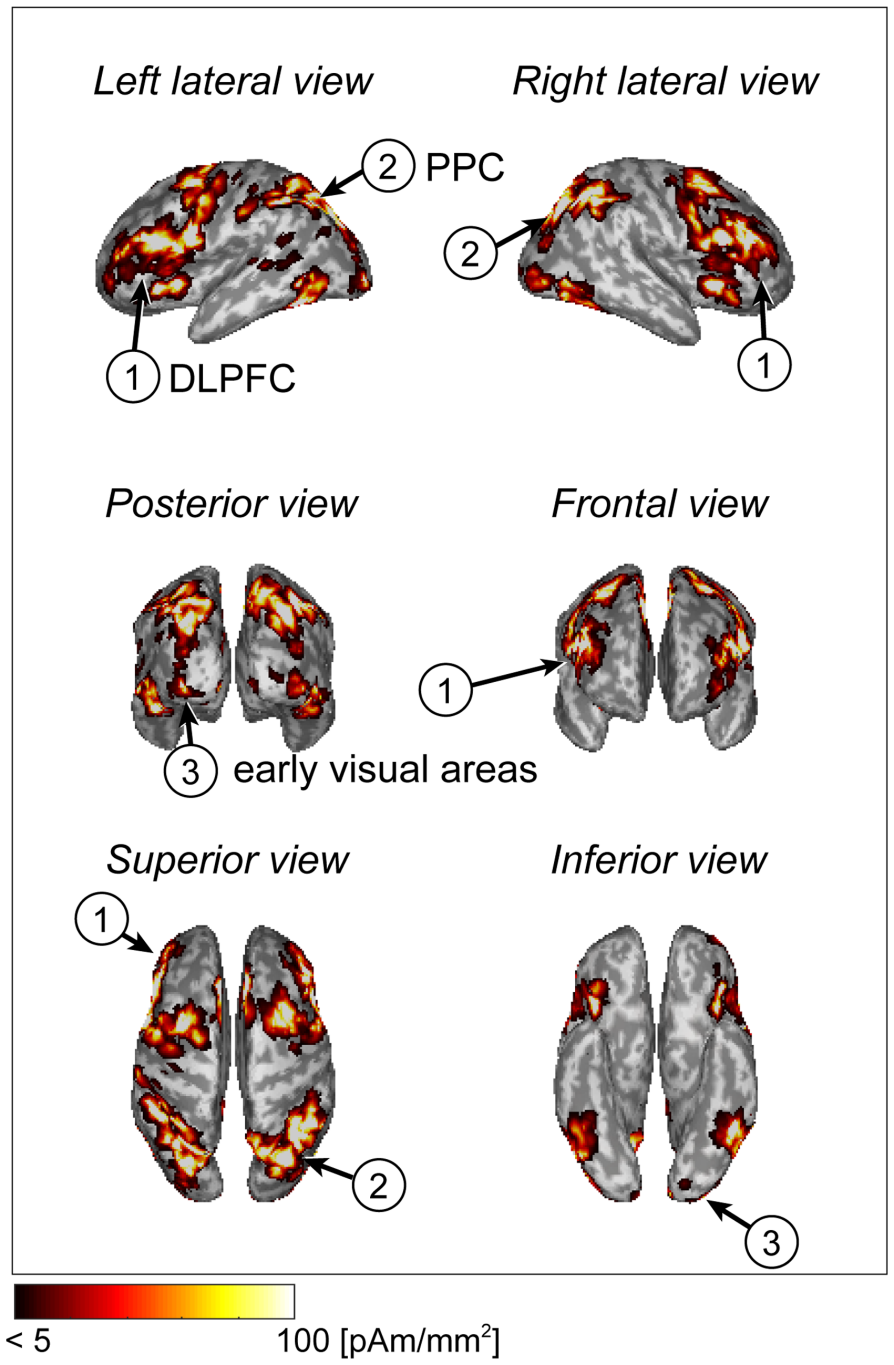
Cortical current distribution using a statistical map generated by Neurosynth (example of a typical subject).

The cortical current in each participant was estimated using the extra-dipole method. We calculated the trial-averaged values from the estimated current densities and plotted the absolute and maximum values on the cortical surface model. The cortical regions of the dorsolateral prefrontal cortex (DLPFC), posterior parietal cortex (PPC), and early visual areas showed large current intensities. These areas are related to visual working memory processes (Figure 3).

We also searched for the maximum current densities across all dipoles on the cortical surface of each participant and calculated the mean values and standard deviations. The values were 133.2 ± 99.9 pAm/mm^2^. In previous electrophysiological studies, the estimated current densities ranged from 25 to 250 pAm/mm^2^(Hämäläinen et al., 1993). The values calculated in this study were within these ranges. We also calculated the mean values of the absolute eye currents from single-trial data. These amplitudes ranged from 0.12 to 53.2 nAm, and these estimated values were similar to those of previous research with respect to the order of magnitude (Katila et al., 1981; Morishige et al., 2014).

**Figure 4:**
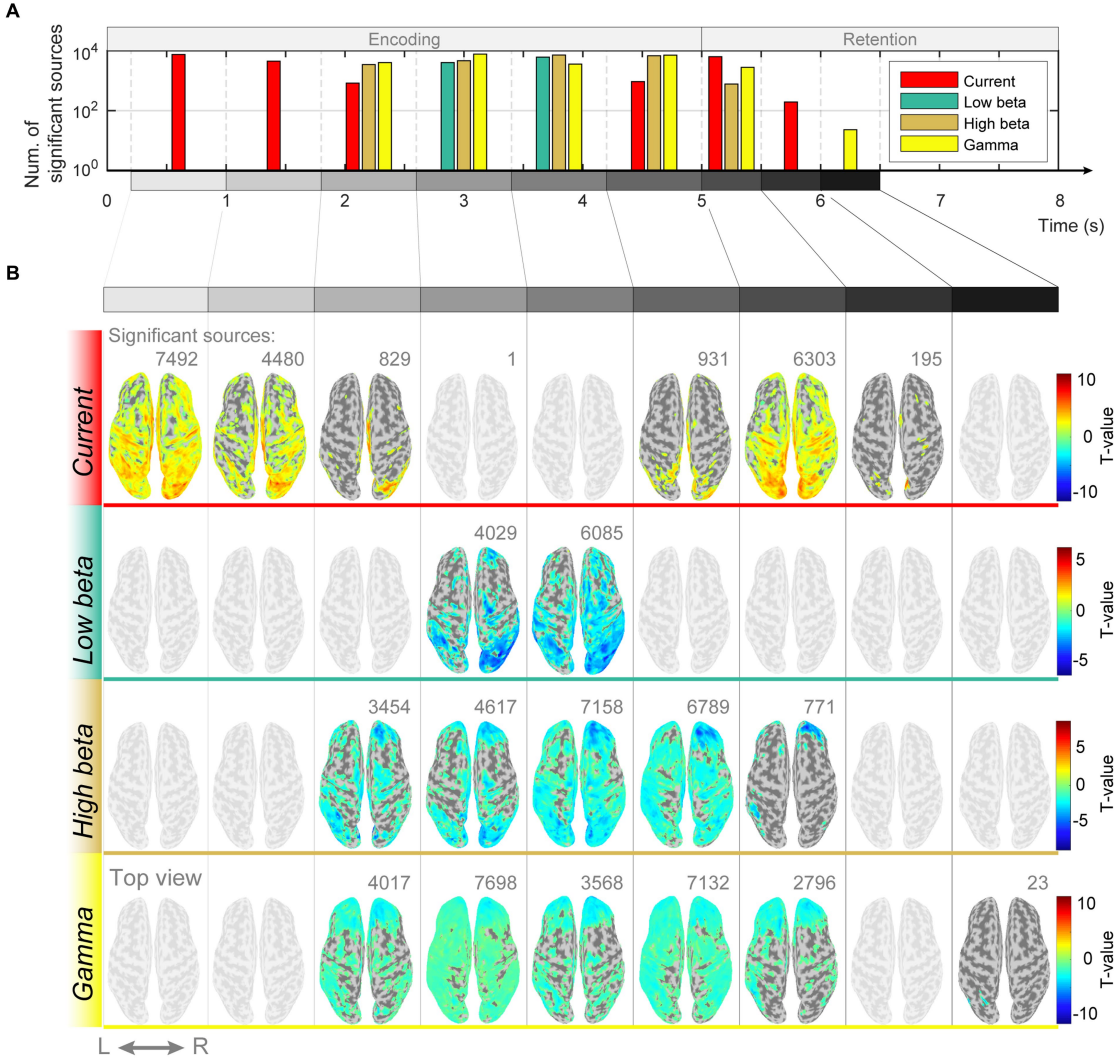
Differences in magnitudes of estimated source currents and power spectral densities between modified n-back and DMTS conditions. **(A)** Number of significant current sources for each subperiod of encoding and retention. **(B)** Significant current source locations on the cortical surface map for the subperiods of encoding and retention.

If memory retention is achieved through sustained neuronal firing patterns, there should be differences in the intensity of the estimated current at each dipole. However, if the function is implemented in periodic patterns, the spectral features of the estimated currents should differ. We examined whether there were differences in the magnitude of the estimated currents in response to different memory loads and found significant differences in the encoding and retention subperiods ([0.2–1.0 s]: *p* = 0.001, FDR-corrected, paired *t*-test; [1.0–1.8 s]: *p* = 0.002, FDR-corrected, paired *t*-test; [1.8–2.6 s]: *p* = 0.01, FDR-corrected, paired *t*-test; [4.2–5.0 s]: *p* = 0.04, FDR-corrected, paired *t*-test; [5.0–5.5 s]: *p* < 0.0001, FDR-corrected, paired *t*-test; [5.5–6.0 s]: *p* = 0.004, FDR-corrected, paired *t*-test; Figures 4A,B). Additionally, spectral features of beta and gamma waves had significant differences in several cortical regions ([1.8–2.6 s]: (high beta) p = 0.001, (gamma) *p* = 0.04, FDR-corrected, paired *t*-test; [2.6–3.4 s]: (low beta) *p* = 0.02, (high beta) *p* = 0.02, (gamma) *p* = 0.04, FDR-corrected, paired *t*-test; [3.4–4.2 s]: (low beta) *p* = 0.02, (high beta) *p* = 0.01, (gamma) *p* = 0.02, FDR-corrected, paired *t*-test; [4.2–5.0 s]: (high beta) *p* = 0.04, (gamma) *p* = 0.03, FDR-corrected, paired *t*-test; [5.0–5.5 s]: (high beta) *p* = 0.01, (gamma) *p* = 0.03, FDR-corrected, paired *t*-test; [6.0–6.5 s]: (gamma) *p* < 0.0001, FDR-corrected, paired *t*-test; [7.0–7.5 s]: (gamma) *p* = 0.04, FDR-corrected, paired *t*-test; Figures 4A,B).

### 3.3. Classification

**Figure 5:**
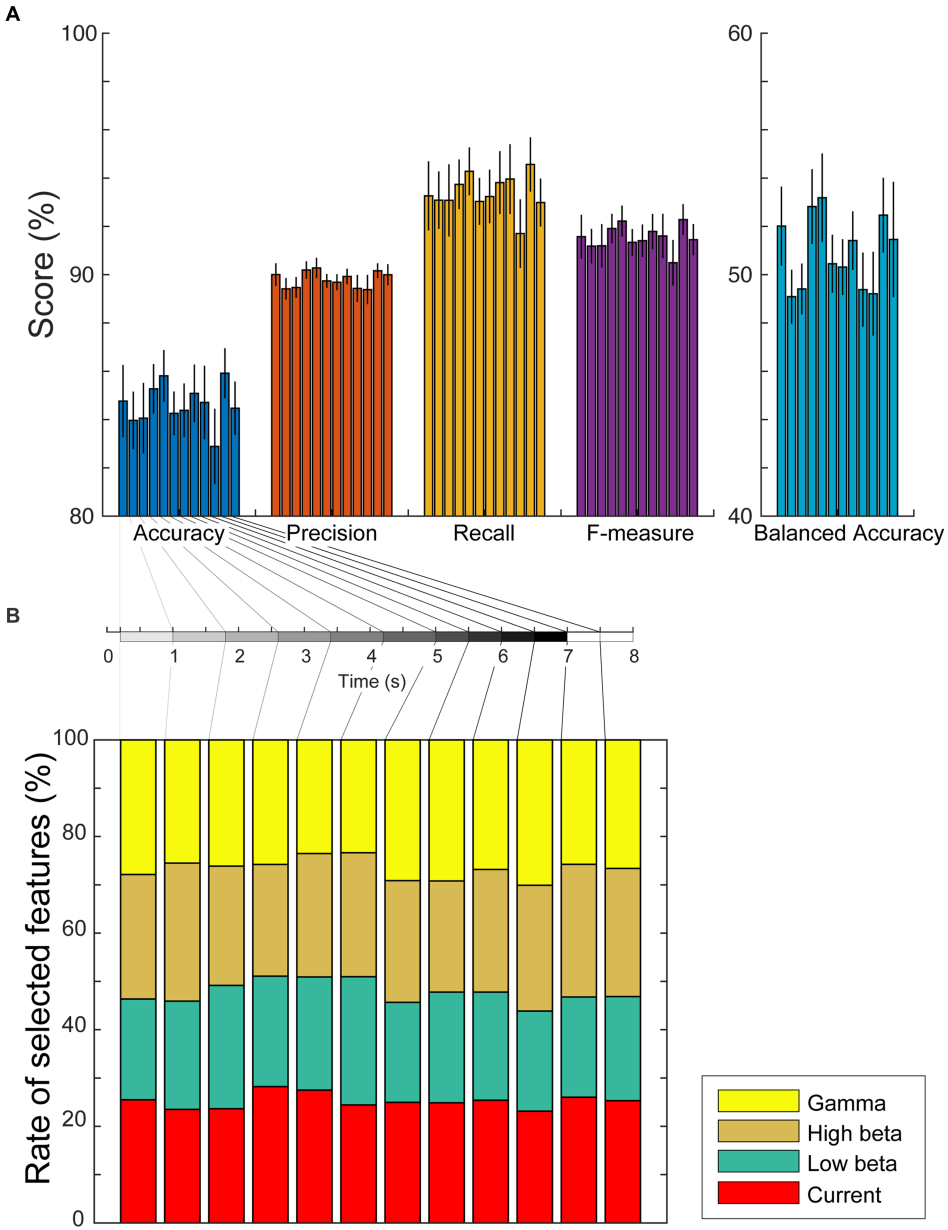
**(A)** Mean values and standard errors of scores (accuracy, precision, recall, F-measure, and balanced accuracy) for each subperiod of encoding and retention using the weighted sparse logistic regression method. **(B)** Ratios of types of selected dipole numbers. We counted the number of times it was selected as a weighted SLR feature for each trial, calculated the mean ratio for each participant, and plotted the average ratios as a stacked bar chart. The rate of selected dipole for currents, low beta, high beta, and gamma waves are shown as red, green, brown, and yellow bars, respectively.

If the estimated cortical currents contain information about visual working memory, the task conditions must be predicted from the currents or power spectra during the encoding and retention periods. Considering the results of the group analysis in the previous subsection, we investigated the representation of working memory task conditions using the current amplitudes and power spectral densities during each encoding/retention subperiod by computing the sum of the absolute current amplitude in a subperiod and the average power spectral densities in each significant frequency band (low beta, high beta, and gamma waves) using Welch’s method. We used weighted sparse logistic regression to reduce the input dimension of the power spectrum densities and classified the trials as modified n-back or DMTS tasks. The classification accuracies in six encoding and six retention subperiods were 84.8 ± 5.1%, 84.0 ± 4.0%, 84.1 ± 5.0%, 85.3 ± 3.5%, 85.8 ± 3.6%, 84.3 ± 3.0%, 84.4 ± 3.7%, 85.1 ± 4.0%, 84.7 ± 5.2%, 82.9 ± 5.3%, 85.9 ± 3.5%, and 84.5 ± 3.7%, respectively (Figure 5A). The precisions were 90.0 ± 1.5%, 89.4 ± 1.4%, 89.5 ± 1.4%, 90.2 ± 1.2%, 90.3 ± 1.4%, 89.7 ± 0.9%, 89.7 ± 1.1%, 89.9 ± 1.0%, 89.4 ± 1.8%, 89.4 ± 2.0%, 90.2 ± 1.0%, and 90.0 ± 1.4%, respectively. The recalls were 93.3 ± 4.9%, 93.1 ± 4.0%, 93.1 ± 5.1%, 93.7 ± 3.5%, 94.3 ± 3.3%, 93.0 ± 3.3%, 93.2 ± 3.8%, 93.8 ± 4.4%, 94.0 ± 4.9%, 91.7 ± 4.8%, 94.6 ± 3.8%, and 93.0 ± 3.3%, respectively. The F-measures were 91.6 ± 3.0%, 91.2 ± 2.4%, 91.2 ± 3.0%, 91.9 ± 2.0%, 92.2 ± 2.1%, 91.3 ± 1.8%, 91.4 ± 2.2%, 91.8 ± 2.4%, 91.6 ± 3.1%, 90.5 ± 3.2%, 92.3 ± 2.1%, and 91.5 ± 2.1%, respectively. The balanced accuracies were 52.0 ± 5.6%, 49.1 ± 3.8%, 49.4 ± 3.6%, 52.8 ± 5.3%, 53.2 ± 6.3%, 50.5 ± 4.1%, 50.3 ± 3.9%, 51.4 ± 4.1%, 49.4 ± 5.2%, 49.2 ± 5.9%, 52.5 ± 5.3%, and 51.5 ± 8.2%, respectively. In total, 72 of all 168 accuracies (= [12 subperiods] × [14 participants]), 64 of 168 precisions, 60 of 168 recalls, 66 of 168 F-measures, and 64 of 168 balanced accuracies reached significance (*p* < 0.05, permutation test, FDR-corrected; Supplementary Tables S2–S6).

To investigate whether the scores differed by time interval, a randomized block design one-way ANOVA was performed. The ANOVA results identified no significant differences among the scores of the subperiods [accuracy: *F*(11,143) = 0.81, *p* = 0.63; precision: *F*(11,143) = 1.29, *p* = 0.33; recall: *F*(11,143) = 0.641, *p* = 0.77; F-measure: *F*(11,143) = 0.79, *p* = 0.65; balanced accuracy: *F*(11,143) = 1.23, *p* = 0.36]. We also used weighted SLR to examine the frequency bands of the features used for identification and found that all types of dipoles, current amplitudes, and low beta, high beta, and gamma waves were selected as discrimination features for each subperiod (Figure 5B).

## 4. Discussion

In this study, we examined the brain mechanisms underlying executive control over memory retention in working memory to determine whether this was a simple persistent spiking or a periodic pattern. We measured the scalp EEG data, while the participants performed modified n-back working memory tasks and estimated the cortical currents from the EEG data by introducing a statistical map generated by Neurosynth as prior information. A group analysis of the cortical current level revealed that both the current amplitudes and power spectra were significantly different between the modified n-back and DMTS conditions. We classified information on the working memory task conditions using the power spectrum of the currents during the encoding and retention periods. Our results indicate that executive control over memory retention may be represented by both current amplitudes and oscillatory representations in the beta and gamma bands over multiple cortical regions that contribute to visual working memory function.

Although group analysis methods are commonly used in the analysis of fMRI data, they have not been previously applied to whole-brain cortical currents estimated from observed data owing to technical difficulties. In this study, using the method by Takeda et al. (2019) in combination with the extra-dipole method (Morishige et al., 2014), eye artifacts can be effectively removed at the current estimation stage and examined using the obtained cortical currents with high temporal–spatial resolution. It is particularly significant that we investigated the changes in brain activities during a short time interval (0.8 s visual cue repetition on encoding period and 3 s retention) of memory encoding and retention by performing a time-frequency analysis with high spatial resolution.

In the original version of n-back task, the overlap between the encoding and retention periods prevented a clear separation of the functional roles of the two for discussion. We revised the experimental paradigm and established separate retentions to allow for a clear separation from the encoding period.

During the retention period, both modified n-back task and the DMTS task required participants to temporarily remember one (or a few) of the stimuli repeatedly presented seven times. When comparing the cortical currents in the modified n-back and DMTS task conditions over the retention period, if there was evidence of behaviors in which working memory was used more strongly during this period under the modified n-back task condition, significant differences in the retention period would be expected; however, there was no evidence of such a behavior. Behavioral performance (success rate) in the modified n-back task condition was lower than that in the DMTS task condition because it only represented the difficulty of encoding. Therefore, the difference between the two groups with respect to working memory should be investigated during the period of encoding rather than retention.

In our experiment, the modified n-back and DMTS tasks were presented randomly without any additional instructions. In the flow of the DMTS task, the same arrows were presented repeatedly. The participant becomes intuitive about the third arrow and is convinced that this is a DMTS task through the presentation of the red stimulus. Therefore, before presenting the first or second stimulus, the participants did not realize that it was a DMTS task or a modified n-back task. In our group analysis, we investigated the differences between the modified n-back and DMTS tasks, so the significant differences in the low/high beta and gamma bands were found in the time intervals from the third subperiod of encoding to the first subperiod of the retention, which were also reasonable results.

Pesonen et al. examined event-related desynchronization (ERD) and event-related synchronization (ERS) responses for targets and non-targets under four different memory load conditions (0-, 1-, 2-, and 3-back) from EEG data (Pesonen et al., 2007). They found that the early-appearing beta rhythm (14–30 Hz) decreased with an increasing memory load. Additionally, the beta rhythms increased under the 0- and 1-back memory load conditions. Our group analysis results correspond to the differences in the power of the beta frequency band calculated from the 2-back and 0-back tasks. Therefore, the finding that beta is significantly negative is consistent with the results of Pesonen et al.

In addition, event-related brain oscillatory responses in the beta frequency range are associated with cognitive processing and motor cortex activity. In the original version of the n-back task, participants were required to respond by pressing a button immediately after the presentation of the visual stimulus. The encoding period of working memory and the period of motor preparation overlap, making it difficult to distinguish between the beta waves originating from both. By contrast, in our modified n-back task, the button was pressed after the retention period. Therefore, the effects of oscillations on motor planning and cognitive memory processes should be discussed separately. Our results suggest that beta oscillations mainly reflect the influence of cognitive and memory processing and that the effect of motor planning is small.

The subperiods with significant differences in gamma oscillations overlapped with those in beta oscillations. It has been hypothesized that gamma and beta oscillations may be synchronized. Lundqvist et al. examined brief bursts of high gamma (50–120 Hz) and high beta (20–35 Hz) oscillations in monkeys (Lundqvist et al., 2018). Beta bursts are associated with suppressing gamma bursts and object information during spiking. Gamma and beta bursting were anti-correlated over time but only at recording sites where spiking carried information about objects to be remembered. The interplay between beta and gamma bursts suggests a potential mechanism for controlling working memory. The relationship between high gamma and high beta oscillations should also be investigated.

Pesonen et al. showed that the magnitude of alpha oscillations decreases with memory load (Krause et al., 2000; Pesonen et al., 2007). However, in this study, no significant differences between the modified n-back and DMTS task conditions were observed in any subperiod of encoding and retention (all subperiods and dipoles of theta and alpha oscillations, *p* > 0.05, FDR-corrected, paired *t*-test). Haegens et al. suggested that alpha oscillations have similar inhibitory roles in sensory-motor areas in DMTS tasks. In general, sensory alpha has been suggested to have inhibitory functions, and it might be that beta has a similar role, but the frequency is shifted upward in the higher-order cortex. Interactions between the mediodorsal thalamus and prefrontal cortex likely produce beta oscillations. Thus, Lundqvist et al. hypothesized that the network between the mediodorsal thalamus and prefrontal cortex might be involved in regulating working memory activity. In contrast, the superficial layers of the prefrontal cortex may contain the contents themselves (Lundqvist et al., 2018). Therefore, there may have been no significant difference between the alpha oscillations of the modified n-back and DMTS conditions in this study. However, there is another possibility that these discrepancies between previous studies and our results may be at least partially explained by different task flows. The original version of the n-back task required a constant memory load because encoding and retention were repeated simultaneously. In contrast, in the modified n-back task used in this study, encoding and retention were sequential and repeated with a short break after each trial. Therefore, the effect of memory load varies among subperiods, and its effect may be relatively small.

The potential increased in the parietal region 300 ms after the visual stimulus presentation. Moreover, it is also known that the potential varies with the magnitude of the memory load (McEvoy, 1998; Segalowitz et al., 2001). The time interval during which the seventh visual stimulus was presented was the time of the greatest memory load in the 2-back task. In the current study, the estimated currents were significantly larger during the time range in which the sixth and seventh visual stimuli were presented, possibly for these reasons. However, the estimated currents were also significantly larger during the encoding subperiods when the first and second visual stimuli were presented. The main reason for this was presumably an imbalance in the number of trials in the modified n-back and DMTS tasks. The modified n-back task had a larger proportion of trials; therefore, the participants tended to expect the modified n-back task to start before each trial began. Because we are not certain if this is the main reason, we should review the observed data to clarify the cause.

Attempts to decode working memory contents have been made by many researchers using various measurement techniques such as neural activities, scalp surface EEG, MEG, and fMRI (Harrison and Tong, 2009; Christophel et al., 2012; Syrjälä et al., 2021). Many studies have reported that periodic components of theta/alpha bandwidths contribute to the representation of memory content and task conditions (Kawasaki et al., 2010; Sauseng et al., 2010; Akiyama et al., 2017) and that beta and gamma bandwidths contribute to their realization (Howard et al., 2003; Lundqvist et al., 2016; Daume et al., 2017; Lundqvist et al., 2018). The ability to classify memory content by using fMRI suggests the presence of specific activity patterns. Although various ways of representing the contents of working memory have been proposed, there are too few methods that discuss them in a unified manner. By combining methods of estimating cortical currents from EEG data and classifying brain information from the estimated currents using the SLR, it is possible to examine brain activity related to working memory with higher temporal and spatial resolutions than that associated with conventional methods. Our results indicate that both persistent neural and oscillatory activities in specific brain regions contribute to the retention of memory task conditions, but both contribute to its realization in a wide range of brain regions.

The time intervals with significant differences varied widely among the participants. Individual differences may be large because of differences in information processing abilities and strategies among participants. Classification may be significant in the subperiods of encoding after the presentation of the first and second visual stimuli. This finding may also be explained by an imbalance in the number of trials required for the modified n-back and DMTS tasks.

In this study, we analyzed the estimated cortical currents only during the encoding and retention periods. However, using our method of analysis, it is also possible to analyze the retrieval periods. Therefore, in future, we would like to clarify how working memory task conditions and their contents are represented not only in the encoding and retention periods but also in the retrieval periods. In addition, we conduct experiments not only on the modified 2-back task but also on the modified 3-back task, which is more difficult. We compute the current amplitudes and power spectra and compare them and classification of correct and incorrect response items in the modified n-back task to confirm that both persistent neural and oscillatory activities are associated with working memory contents and loads.

## Data availability statement

The raw data supporting the conclusions of this article will be made available by the authors, without undue reservation.

## Ethics statement

The studies involving humans were approved by Ethics Committee of Toyama Prefectural University. The studies were conducted in accordance with the local legislation and institutional requirements. The participants provided their written informed consent to participate in this study.

## Author contributions

HT, KI, MK, and K-iM designed the experiments. SY and K-iM performed the acquisition, wrote the code for data analysis, and wrote the manuscript. All authors contributed to the article and approved the submitted version.

## Footnotes

^1^https://neurosynth.org
