# Supplementary material for: Group analysis and classification of working memory task conditions using electroencephalogram cortical currents during an n-back task
Source: Front Neurosci. 2023 Oct 24;17:1222749. doi: 10.3389/fnins.2023.1222749 (PMC10627866; doi:10.3389/fnins.2023.1222749)
Supplement: Supplementary file 1 [file Data_Sheet_1.PDF]

# Supplementary information

## Group analysis and classification of working memory task conditions using electroencephalogram cortical currents during an n-back task

Shinnosuke Yoshiiwa[1†], Hironobu Takano[2], Keisuke Ido[3], Mitsuo Kawato[2,4], Ken-ichi Morishige[2,5*†]

[1] *Graduate School of Engineering, Toyama Prefectural University, Toyama, Japan*

[2] *Department of Intelligent Robotics, Toyama Prefectural University, Toyama, Japan*

[3] *Center of Liberal Arts and Science, Toyama Prefectural University, Toyama, Japan*

[4] *Brain Information Communication Research Laboratory Group, Advanced Telecommunications Research Institute International, Kyoto, Japan*

[5] *Neural Information Analysis Laboratories, Advanced Telecommunications Research Institute International, Kyoto, Japan*

*** Correspondence:**

Ken-ichi Morishige

kmorishi@pu-toyama.ac.jp

† These authors contributed equally to this work and share first authorship.

## 1. Trial number

Supplementary Table S1

| Participant ID | Observed trial number | Trial number after removal of EOG and large artifacts | Trial number after removal of incorrect responses |
|---|---|---|---|
| P01 | 160 | 139 | 135 |
| P02 | 160 | 159 | 152 |
| P03 | 160 | 145 | 141 |
| P04 | 160 | 150 | 147 |
| P05 | 160 | 135 | 129 |
| P06 | 160 | 159 | 149 |
| P07 | 160 | 152 | 144 |
| P08 | 160 | 129 | 89 |
| P09 | 160 | 160 | 145 |
| P10 | 160 | 84 | 54 |
| P11 | 160 | 138 | 129 |
| P12 | 160 | 154 | 145 |
| P13 | 160 | 85 | 67 |
| P14 | 160 | 160 | 158 |

## 2. Results of Permutation test

Supplementary Table S2 (Accuracy, one-sided test, FDR-corrected)

| **Participant ID** | **0.2–1.0 s** | **1.0–1.8 s** | **1.8–2.6 s** | **2.6–3.4 s** | **3.4–4.2 s** | **4.2–5.0 s** | **5.0–5.5 s** | **5.5–6.0 s** | **6.0–6.5 s** | **6.5–7.0 s** | **7.0–7.5 s** | **7.5–8.0 s** |
|---|---|---|---|---|---|---|---|---|---|---|---|---|
| P01 | 88.89 | 78.52 | 88.89 | 82.22 | 85.93 | 82.22 | 89.63 | 89.63 | 88.89 | 88.15 | 89.63 | 80.74 |
| P02 | 78.95 | 83.55 | 84.87 | 81.58 | 86.84 | 84.21 | 83.55 | 84.87 | 86.84 | 78.95 | 87.50 | 82.89 |
| P03 | 90.07 * | 85.11 * | 87.23 * | 82.27 * | 86.52 * | 84.40 * | 79.43 * | 86.52 * | 90.07 * | 89.36 * | 82.27 * | 85.11 * |
| P04 | 80.95 * | 82.99 * | 82.99 * | 87.76 * | 82.99 * | 89.80 * | 85.03 * | 88.44 * | 83.67 * | 80.95 * | 87.76 * | 80.27 * |
| P05 | 88.37 | 88.37 | 89.15 | 85.27 † | 89.15 | 80.62 | 82.17 | 82.17 | 86.05 | 75.19 | 81.40 | 80.62 |
| P06 | 84.56 | 80.54 | 83.89 | 86.58 | 82.55 | 84.56 | 81.88 | 76.51 | 86.58 | 86.58 | 81.88 | 78.52 |
| P07 | 79.17 | 85.42 † | 86.81 † | 86.11 † | 84.72 † | 82.64 † | 86.11 † | 83.33 † | 84.03 † | 84.72 † | 88.19 † | 83.33 † |
| P08 | 84.27 * | 86.52 * | 78.65 * | 79.78 * | 91.01 * | 80.90 * | 85.39 * | 83.15 * | 77.53 * | 77.53 * | 87.64 * | 89.89 * |
| P09 | 85.52 | 80.69 | 77.93 | 85.52 | 78.62 | 84.14 | 86.21 | 84.14 | 73.79 | 77.24 | 80.00 | 87.59 |
| P10 | 90.74 * | 90.74 * | 87.04 * | 90.74 * | 88.89 * | 90.74 * | 85.19 * | 90.74 * | 88.89 * | 88.89 * | 88.89 * | 88.89 * |
| P11 | 90.70 | 89.92 | 90.70 | 89.92 | 89.15 | 84.50 | 92.25 † | 90.70 | 89.92 | 89.92 | 90.70 | 89.92 |
| P12 | 88.28 * | 81.38 * | 86.21 * | 80.69 * | 88.97 * | 85.52 * | 80.00 * | 80.69 * | 80.69 * | 81.38 * | 84.83 * | 83.45 * |
| P13 | 74.63 * | 77.61 * | 74.63 * | 88.06 * | 80.60 * | 80.60 * | 79.10 * | 83.58 * | 79.10 * | 76.12 * | 83.58 * | 86.57 * |
| P14 | 81.65 | 84.18 | 77.85 | 87.34 | 85.44 | 84.81 | 85.44 | 86.71 | 89.87 | 85.44 | 88.61 | 84.81 |

† $p < 0.1$, *$p < 0.05$

Supplementary Table S3 (Precision, one-sided test, FDR-corrected)

| **Participant ID** | **0.2–1.0 s** | **1.0–1.8 s** | **1.8–2.6 s** | **2.6–3.4 s** | **3.4–4.2 s** | **4.2–5.0 s** | **5.0–5.5 s** | **5.5–6.0 s** | **6.0–6.5 s** | **6.5–7.0 s** | **7.0–7.5 s** | **7.5–8.0 s** |
|---|---|---|---|---|---|---|---|---|---|---|---|---|
| P01 | 90.23 | 89.08 | 90.23 | 89.52 | 90.55 | 89.52 | 90.30 | 90.30 | 90.23 | 90.15 | 91.54 | 90.00 |
| P02 | 88.81 * | 90.51 * | 88.97 * | 89.71 * | 91.43 * | 89.44 * | 89.93 * | 89.51 * | 89.19 * | 88.81 * | 89.26 * | 88.73 * |
| P03 | 91.85 * | 90.15 | 90.37 | 89.84 | 90.91 | 90.08 | 88.89 | 90.30 | 92.48 * | 93.08 * | 90.48 | 91.41 † |
| P04 | 88.81 | 89.63 | 89.05 | 91.91 * | 90.84 | 89.80 | 89.86 | 89.66 | 89.13 | 88.81 | 89.58 | 89.31 |
| P05 | 90.98 | 89.06 | 89.15 | 90.68 | 89.76 | 88.79 | 88.98 | 89.66 | 88.80 | 87.39 | 88.89 | 88.14 |
| P06 | 90.44 | 88.81 | 89.78 | 90.65 | 89.05 | 89.86 | 89.55 | 87.69 | 90.65 | 90.07 | 88.97 | 87.97 |
| P07 | 88.98 * | 89.13 * | 90.44 * | 89.21 * | 89.63 * | 88.81 * | 90.37 * | 90.70 * | 89.55 * | 89.63 * | 91.18 * | 88.89 * |
| P08 | 90.12 * | 90.36 * | 88.46 * | 88.61 * | 92.77 * | 88.75 * | 90.24 * | 91.03 * | 87.34 * | 87.34 * | 91.46 * | 92.68 * |
| P09 | 90.91 | 89.15 | 88.19 | 90.30 | 87.69 | 90.77 | 88.65 | 88.97 | 86.99 | 88.10 | 89.68 | 90.51 |
| P10 | 90.74 * | 90.74 * | 90.38 * | 90.74 * | 90.57 * | 90.74 * | 90.20 * | 90.74 * | 90.57 * | 90.57 * | 90.57 * | 90.57 * |
| P11 | 91.41 | 91.34 | 91.41 | 92.00 | 91.27 | 90.83 | 92.19 | 91.41 | 91.34 | 91.34 | 91.41 | 91.34 |
| P12 | 91.24 | 88.72 | 91.04 | 88.64 | 91.30 | 90.98 | 88.55 | 89.84 | 89.84 | 90.55 | 89.13 | 88.97 |
| P13 | 85.96 * | 85.25 * | 85.96 * | 89.06 * | 88.14 * | 88.14 * | 87.93 * | 88.52 * | 85.48 * | 85.00 * | 89.83 * | 91.53 * |
| P14 | 89.58 | 89.86 | 89.13 | 91.84 | 90.00 | 89.93 | 90.00 | 90.67 | 90.45 | 90.54 | 90.32 | 89.93 |

† $p < 0.1$, *$p < 0.05$

Supplementary Table S4 (Recall, one-sided test, FDR-corrected)

| **Participant ID** | **0.2–1.0 s** | **1.0–1.8 s** | **1.8–2.6 s** | **2.6–3.4 s** | **3.4–4.2 s** | **4.2–5.0 s** | **5.0–5.5 s** | **5.5–6.0 s** | **6.0–6.5 s** | **6.5–7.0 s** | **7.0–7.5 s** | **7.5–8.0 s** |
|---|---|---|---|---|---|---|---|---|---|---|---|---|
| P01 | 98.36 | 86.89 | 98.36 | 90.98 | 94.26 | 90.98 | 99.18 | 99.18 | 98.36 | 97.54 | 97.54 | 88.52 |
| P02 | 87.50 | 91.18 | 94.85 | 89.71 | 94.12 | 93.38 | 91.91 | 94.12 | 97.06 | 87.50 | 97.79 | 92.65 |
| P03 | 97.64 * | 93.70 * | 96.06 * | 90.55 * | 94.49 * | 92.91 * | 88.19 * | 95.28 * | 96.85 * | 95.28 * | 89.76 * | 92.13 * |
| P04 | 90.15 * | 91.67 * | 92.42 * | 94.70 * | 90.15 * | 100.00 * | 93.94 * | 98.48 * | 93.18 * | 90.15 * | 97.73 * | 88.64 * |
| P05 | 96.52 | 99.13 | 100.00 | 93.04 | 99.13 | 89.57 | 91.30 | 90.43 | 96.52 | 84.35 | 90.43 | 90.43 |
| P06 | 92.48 | 89.47 | 92.48 | 94.74 | 91.73 | 93.23 | 90.23 | 85.71 | 94.74 | 95.49 | 90.98 | 87.97 |
| P07 | 87.60 | 95.35 | 95.35 | 96.12 | 93.80 | 92.25 | 94.57 | 90.70 | 93.02 | 93.80 | 96.12 | 93.02 |
| P08 | 92.41 * | 94.94 * | 87.34 * | 88.61 * | 97.47 * | 89.87 * | 93.67 * | 89.87 * | 87.34 * | 87.34 * | 94.94 * | 96.20 * |
| P09 | 93.02 | 89.15 | 86.82 | 93.80 | 88.37 | 91.47 | 96.90 | 93.80 | 82.95 | 86.05 | 87.60 | 96.12 |
| P10 | 100.00 * | 100.00 * | 95.92 * | 100.00 * | 97.96 * | 100.00 * | 93.88 * | 100.00 * | 97.96 * | 97.96 * | 97.96 * | 97.96 * |
| P11 | 99.15 | 98.31 | 99.15 | 97.46 | 97.46 | 92.37 | 100.00 † | 99.15 | 98.31 | 98.31 | 99.15 | 98.31 |
| P12 | 96.15 | 90.77 | 93.85 | 90.00 | 96.92 | 93.08 | 89.23 | 88.46 | 88.46 | 88.46 | 94.62 | 93.08 |
| P13 | 84.48 * | 89.66 * | 84.48 * | 98.28 * | 89.66 * | 89.66 * | 87.93 * | 93.10 * | 91.38 * | 87.93 * | 91.38 * | 93.10 * |
| P14 | 90.21 | 93.01 | 86.01 | 94.41 | 94.41 | 93.71 | 94.41 | 95.10 | 99.30 | 93.71 | 97.90 | 93.71 |

† $p < 0.1$, *$p < 0.05$

Supplementary Table S5 (F-measure, one-sided test, FDR-corrected)

| **Participant ID** | **0.2–1.0 s** | **1.0–1.8 s** | **1.8–2.6 s** | **2.6–3.4 s** | **3.4–4.2 s** | **4.2–5.0 s** | **5.0–5.5 s** | **5.5–6.0 s** | **6.0–6.5 s** | **6.5–7.0 s** | **7.0–7.5 s** | **7.5–8.0 s** |
|---|---|---|---|---|---|---|---|---|---|---|---|---|
| P01 | 94.12 | 87.97 | 94.12 | 90.24 | 92.37 | 90.24 | 94.53 | 94.53 | 94.12 | 93.70 | 94.44 | 89.26 |
| P02 | 88.15 | 90.84 | 91.81 | 89.71 | 92.75 | 91.37 | 90.91 | 91.76 | 92.96 | 88.15 | 93.33 | 90.65 |
| P03 | 94.66 * | 91.89 * | 93.13 * | 90.20 * | 92.66 * | 91.47 * | 88.54 * | 92.72 * | 94.62 * | 94.16 * | 90.12 * | 91.76 * |
| P04 | 89.47 * | 90.64 * | 90.71 * | 93.28 * | 90.49 * | 94.62 * | 91.85 * | 93.86 * | 91.11 * | 89.47 * | 93.48 * | 88.97 * |
| P05 | 93.67 | 93.83 | 94.26 | 91.85 † | 94.21 | 89.18 | 90.13 | 90.04 | 92.50 | 85.84 | 89.66 | 89.27 |
| P06 | 91.45 | 89.14 | 91.11 | 92.65 | 90.37 | 91.51 | 89.89 | 86.69 | 92.65 | 92.70 | 89.96 | 87.97 |
| P07 | 88.28 | 92.13 | 92.83 | 92.54 | 91.67 | 90.49 | 92.42 | 90.70 | 91.25 | 91.67 | 93.58 | 90.91 |
| P08 | 91.25 * | 92.59 * | 87.90 † | 88.61 † | 95.06 * | 89.31 † | 91.93 * | 90.45 † | 87.34 † | 87.34 † | 93.17 * | 94.41 * |
| P09 | 91.95 | 89.15 | 87.50 | 92.02 | 88.03 | 91.12 | 92.59 | 91.32 | 84.92 | 87.06 | 88.63 | 93.23 |
| P10 | 95.15 * | 95.15 * | 93.07 * | 95.15 * | 94.12 * | 95.15 * | 92.00 * | 95.15 * | 94.12 * | 94.12 * | 94.12 * | 94.12 * |
| P11 | 95.12 | 94.69 | 95.12 | 94.65 | 94.26 | 91.60 | 95.93 † | 95.12 | 94.69 | 94.69 | 95.12 | 94.69 |
| P12 | 93.63 * | 89.73 * | 92.42 * | 89.31 * | 94.03 * | 92.02 * | 88.89 * | 89.15 * | 89.15 * | 89.49 * | 91.79 * | 90.98 * |
| P13 | 85.22 * | 87.39 * | 85.22 * | 93.44 * | 88.89 * | 88.89 * | 87.93 * | 90.76 * | 88.33 * | 86.44 * | 90.60 * | 92.31 * |
| P14 | 89.90 | 91.41 | 87.54 | 93.10 | 92.15 | 91.78 | 92.15 | 92.83 | 94.67 | 92.10 | 93.96 | 91.78 |

† $p < 0.1$, *$p < 0.05$

Supplementary Table S6 (Balanced Accuracy, one-sided test, FDR-corrected)

| **Participant ID** | **0.2–1.0 s** | **1.0–1.8 s** | **1.8–2.6 s** | **2.6–3.4 s** | **3.4–4.2 s** | **4.2–5.0 s** | **5.0–5.5 s** | **5.5–6.0 s** | **6.0–6.5 s** | **6.5–7.0 s** | **7.0–7.5 s** | **7.5–8.0 s** |
|---|---|---|---|---|---|---|---|---|---|---|---|---|
| P01 | 49.18 | 43.44 | 49.18 | 45.49 | 50.98 | 45.49 | 49.59 | 49.59 | 49.18 | 48.77 | 56.46 | 48.11 |
| P02 | 46.88 * | 54.96 * | 47.43 * | 51.10 * | 59.56 * | 49.82 * | 52.21 * | 50.18 * | 48.53 * | 46.88 * | 48.90 * | 46.32 * |
| P03 | 59.53 * | 50.42 | 51.60 | 48.85 | 54.39 | 50.03 | 44.09 | 51.21 | 62.71 * | 65.49 * | 52.02 | 56.78 † |
| P04 | 45.08 | 49.17 | 46.21 | 60.68 * | 55.08 | 50.00 | 50.30 | 49.24 | 46.59 | 45.08 | 48.86 | 47.65 |
| P05 | 58.98 | 49.57 | 50.00 | 57.24 | 53.14 | 48.35 | 49.22 | 52.36 | 48.26 | 42.17 | 48.79 | 45.22 |
| P06 | 55.62 | 47.86 | 52.49 | 56.74 | 48.99 | 52.87 | 51.36 | 42.86 | 56.74 | 53.99 | 48.61 | 43.98 |
| P07 | 47.13 * | 47.67 * | 54.34 * | 48.06 * | 50.23 * | 46.12 * | 53.95 * | 55.35 * | 49.84 * | 50.23 * | 58.06 * | 46.51 * |
| P08 | 56.20 * | 57.47 * | 48.67 * | 49.30 * | 68.73 * | 49.94 * | 56.84 * | 59.94 * | 43.67 * | 43.67 * | 62.47 * | 68.10 * |
| P09 | 59.01 | 50.82 | 46.54 | 56.27 | 44.19 | 58.24 | 48.45 | 50.02 | 41.47 | 46.15 | 53.17 | 57.44 |
| P10 | 50.00 * | 50.00 * | 47.96 * | 50.00 * | 48.98 * | 50.00 * | 46.94 * | 50.00 * | 48.98 * | 48.98 * | 48.98 * | 48.98 * |
| P11 | 49.58 | 49.15 | 49.58 | 53.27 | 48.73 | 46.19 | 54.55 | 49.58 | 49.15 | 49.15 | 49.58 | 49.15 |
| P12 | 58.08 | 45.38 | 56.92 | 45.00 | 58.46 | 56.54 | 44.62 | 50.90 | 50.90 | 54.23 | 47.31 | 46.54 |
| P13 | 47.80 * | 44.83 * | 47.80 * | 60.25 * | 55.94 * | 55.94 * | 55.08 * | 57.66 * | 45.69 * | 43.97 * | 62.36 * | 68.77 * |
| P14 | 45.10 | 46.50 | 43.01 | 57.20 | 47.20 | 46.85 | 47.20 | 50.89 | 49.65 | 50.19 | 48.95 | 46.85 |

† $p < 0.1$, *$p < 0.05$
